# Supplementary material for: The association between symptoms of gastroesophageal reflux disease and perceived stress: A countrywide study of Sri Lanka
Source: PLoS One. 2023 Nov 9;18(11):e0294135. doi: 10.1371/journal.pone.0294135 (PMC10635461; doi:10.1371/journal.pone.0294135)
Supplement: Attachment — Submitted filename: Response to Reviewers.docx [file pone.0294135.s001.docx]

9^th^ July 9, 2023

Dear Editors and Reviwers,

Thank you very much for the comprehensive review of the paper. It helped to greatly enhance the research idea. We have done the required revisions. This document shows the point-by-point responses to the corrections and questions posed by both editors and reviewers. My responses are given in red text for ease of reading. Please let me know if there are any mistakes I can revise if not done so far.

Sincerely

Nilanka Wickramasinghe

When submitting your revision, we need you to address these additional requirements.

1. Please ensure that your manuscript meets PLOS ONE's style requirements, including those for file naming. The PLOS ONE style templates can be found at

<https://journals.plos.org/plosone/s/file?id=wjVg/PLOSOne_formatting_sample_main_body.pdf> and 
<https://journals.plos.org/plosone/s/file?id=ba62/PLOSOne_formatting_sample_title_authors_affiliations.pdf>

Done

2. We note that you have indicated that data from this study are available upon request. PLOS only allows data to be available upon request if there are legal or ethical restrictions on sharing data publicly. For more information on unacceptable data access restrictions, please see <http://journals.plos.org/plosone/s/data-availability#loc-unacceptable-data-access-restrictions>.

In your revised cover letter, please address the following prompts:

a) If there are ethical or legal restrictions on sharing a de-identified data set, please explain them in detail (e.g., data contain potentially sensitive information, data are owned by a third-party organization, etc.) and who has imposed them (e.g., an ethics committee). Please also provide contact information for a data access committee, ethics committee, or other institutional body to which data requests may be sent.

b) If there are no restrictions, please upload the minimal anonymized data set necessary to replicate your study findings as either Supporting Information files or to a stable, public repository and provide us with the relevant URLs, DOIs, or accession numbers. For a list of acceptable repositories, please see <http://journals.plos.org/plosone/s/data-availability#loc-recommended-repositories>.

We will update your Data Availability statement on your behalf to reflect the information you provide.

We have uploaded the data set to a repository as requested.

Please see data set <https://doi.org/10.7910/DVN/WGSNWT> available at HARVARD Dataverse

<https://dataverse.harvard.edu/file.xhtml?fileId=7236399&version=DRAFT>

Dataset Citation

wickramasinghe, nilanka, 2023, "The association between symptoms of Gastroesophageal Reflux Disease and stress: A countrywide study of Sri Lanka", <https://doi.org/10.7910/DVN/WGSNWT>, Harvard Dataverse, DRAFT VERSION, UNF:6:MUMqzMNc1gcPWqZzivvsiA== [fileUNF]

File Citation

wickramasinghe, nilanka, 2023, "The association between symptoms of Gastroesophageal Reflux Disease and stress: A countrywide study of Sri Lanka", <https://doi.org/10.7910/DVN/WGSNWT>, Harvard Dataverse, DRAFT VERSION; DataforstressinGERDpaper.tab [fileName], UNF:6:MUMqzMNc1gcPWqZzivvsiA== [fileUNF]

3. Please upload a new copy of Figure 1 as the detail is not clear. Please follow the link for more information: 
<https://blogs.plos.org/plos/2019/06/looking-good-tips-for-creating-your-plos-figures-graphics/>
<https://blogs.plos.org/plos/2019/06/looking-good-tips-for-creating-your-plos-figures-graphics/>

Done

4. Please include captions for your Supporting Information files at the end of your manuscript, and update any in-text citations to match accordingly. Please see our Supporting Information guidelines for more information: <http://journals.plos.org/plosone/s/supporting-information>.

Reviewer's Responses to Questions

**Comments to the Author**

1. Is the manuscript technically sound, and do the data support the conclusions?

The manuscript must describe a technically sound piece of scientific research with data that supports the conclusions. Experiments must have been conducted rigorously, with appropriate controls, replication, and sample sizes. The conclusions must be drawn appropriately based on the data presented.

Reviewer #1: Yes

Reviewer #2: Yes

2. Has the statistical analysis been performed appropriately and rigorously?

Reviewer #1: Yes

Reviewer #2: Yes

3. Have the authors made all data underlying the findings in their manuscript fully available?

The [PLOS Data policy](http://www.plosone.org/static/policies.action#sharing) requires authors to make all data underlying the findings described in their manuscript fully available without restriction, with rare exception (please refer to the Data Availability Statement in the manuscript PDF file). The data should be provided as part of the manuscript or its supporting information, or deposited to a public repository. For example, in addition to summary statistics, the data points behind means, medians and variance measures should be available. If there are restrictions on publicly sharing data—e.g. participant privacy or use of data from a third party—those must be specified.

Reviewer #1: No - data shared

Reviewer #2: Yes

4. Is the manuscript presented in an intelligible fashion and written in standard English?

PLOS ONE does not copyedit accepted manuscripts, so the language in submitted articles must be clear, correct, and unambiguous. Any typographical or grammatical errors should be corrected at revision, so please note any specific errors here.

Reviewer #1: Yes

Reviewer #2: Yes

5. Review Comments to the Author

Please use the space provided to explain your answers to the questions above. You may also include additional comments for the author, including concerns about dual publication, research ethics, or publication ethics. (Please upload your review as an attachment if it exceeds 20,000 characters)

Reviewer #1: Dear Authors,

It was a pleasure to read your work titled “The association between Gastroesophageal Reflux Disease and stress: A countrywide study of Sri Lanka”.

Overall, a good study with clinical implications. Few concerns


Major

As pointed out by the authors, this is a study on association of various symptoms to the presence/degree of stress. Not necessarily GERD as the study participants did not undergo any formal testing to evaluate for the presence of true evidence of pathological acid reflux. Many of these symptoms can be seen in GERD by are also shared by other etiologies such as functional dyspepsia, functional heartburn, reflux hypersensitivity to name a few. As such, I recommend modifying the title and discussion to reflect this.

Revised title as recommended.

“The association between symptoms of Gastroesophageal Reflux Disease and perceived stress: A countrywide study of Sri Lanka”

Edits made for discussion section as well.

Minor

- Overall, there are multiple long, complex sentences throughout this manuscript. Some have grammatical errors too. This forces the reader stop and retrace the sentence to understand the discussion. I would recommend revising to make the flow smooth and easy on the reader.

We have revised accordingly with use of expert help and software to reduce complex sentences and grammar errors.

- Results section is a little confusing and feels scattered

We have rearranged the results section with advice given by the reviewer.

- Lines 163 to 166. Unclear what the authors are conveying here.

The lines 163 to 166 were rewritten as given below.

“When the score obtained for GERD was correlated with the PSS score for the total population, there was a significant correlation in the total population (R 0.242, *p*<0.001). When the same correlation was done for the subgroups of GERD and controls as seen in Figure 1, a higher correlation was found for those with GERD, (R 0.256 and 0.137 respectively), though results were statistically significant (*p*<0.001) for both GERD and controls.”

- Lines 187 to 189. Do the authors mean "patients with chest pain reported higher stress compared to those without chest pain, but did not reach statistical significance". This sentence is difficult to follow.

Yes. We have rewritten as the reviewer has suggested.

“Patients with chest pain reported higher stress levels than those without, though the result was not statistically significant. (48.4% and 57.9%, Chi-square test, *p*= 0.124).”

- Discussion
  Line 250. "This pattern is observed in our results as well as seen in Table 3." I would elaborate this a little and clarify.

It’s a line erronousely added. We changed from line 241 to 251 it to fit the reviewer’s comment at the very beginning regarding the GERD definition.

“A similar study conducted in Saudi Arabia found that GERD symptoms were significantly (p < 0.05) associated with high perceived stress (OR = 1.30, 95% CI: 1.01–1.44) which is compatible with our results. [31] Furthermore, another study reported that 60% of GERD patients perceived increased symptoms during stressful times. [32] This is compatible with our results which show aggravation of symptoms in 41.8% of subjects during periods of stress.

A previous study has reported a significantly lower level of anxiety, in diagnosed patients with GERD compared to functional heartburn [33] A prospective study in Sweden also found that anxiety was a risk factor for developing functional dyspepsia, but not GERD symptoms. [34] According to these studies, increased levels of anxiety are more likely to be associated with GERD symptoms due to functional heartburn than true GERD. However, since our study did not do pH impedance testing we cannot show the difference in stress levels between subjects with functional disorders and those objectively diagnosed with GERD.”

- Lines 273 to 276. The idea is conveyed but the sentence should be revised.

  "Increased anxiety and stress levels are postulated to affect the GI tract and esophagus in many ways, subsequently triggering true GERD, as well as other conditions which rise to similar symptoms such as reflux hypersensitivity and functional heartburn"

Thank you for revising and making the sentence more understandable. We added the sentence revised by the reviewer.

- Line 316 introduces the abbreviation - FGID without expanding/explaining this. Please expand.

Corrected.

Mucosal barrier dysfunction and increased permeability of the gastrointestinal mucosa are also said to be part of the pathophysiology for Functional Gastrointestinal Disorders (FGIDs).

- Line 326 This sentence is incomplete

  "The current study also identified some associated factors such as younger age, being single, and a low education status" -- Associated with what should be clarified.

Corrected.

The current study also identified some associated factors such as younger age, being single, and a low education status with symptoms of GERD. (Table 2).

- Lines 330 to 334. I think this can be made more succinct.

We have edited the lines as given below

“Studies have shown that anxiety, depression, and other mood disorders increase the odds of developing GERD-related symptoms significantly.[71] [71] In this community-based study, we did not assess such psychiatric conditions due to practical difficulties.”

Reviewer #2: Overall, a well written manuscript on a relevant topic. here are my comments -

1. One fundamental issue with the definition of GERD is based on symptomatology and may encompass several patients with functional dyspepsia and other esophageal motility disorder. This brings the idea of stress directly related to GERD into question. Further detailed explanation of association of symptomatology and perceived stress level would be helpful.

The discussion is amended and edited according to the reviewer comments

We have also changed the title and discussion related to “association of stress to GERD symptoms” as opposed to just “GERD”

2. Provide further explanation regarding lack of symptomatic improvement with GERD medications (what medications were tried and for what duration) in patients with high levels of stress.

The methodology section was revised to include the following.

“The participants were questioned on whether they used any type of proton pump inhibitors (using commonly used generic and trade names to recall), at least once, during the past three months.

They were also questioned on whether the use of PPIs for heartburn, during the past three months, had “no GERD symptom relief” or “achieved GERD symptom relief.”

While revising your submission, please upload your figure files to the Preflight Analysis and Conversion Engine (PACE) digital diagnostic tool, <https://pacev2.apexcovantage.com/>. PACE helps ensure that figures meet PLOS requirements. To use PACE, you must first register as a user. Registration is free. Then, login and navigate to the UPLOAD tab, where you will find detailed instructions on how to use the tool. If you encounter any issues or have any questions when using PACE, please email PLOS at [figures@plos.org](mailto:figures@plos.org). Please note that Supporting Information files do not need this step.
